# Study on the gas desorption law and indicator influencing factors of fixed-size coal samples

**DOI:** 10.1038/s41598-019-53211-4

**Published:** 2019-11-20

**Authors:** Lei Li, Zhongguang Sun, Fakai Wang, Kaizhi Zhang

**Affiliations:** 1 State Key Laboratory of Coal Mine Disaster Dynamics and Control, College of Resources and Environmental Science, Chongqing University, Chongqing, 400044 China; 2 Chongqing Research Institute of China Coal Technology and Engineering Group Crop., Chongqing, 400037 China; 3 College of Mining Engineering, Guizhou Institute of Technology, Guiyang, 550000 China

**Keywords:** Natural hazards, Civil engineering

## Abstract

The prediction of dangerous hazards in working faces is an important link to prevent coal and gas outbursts. Improving the accuracy of predictive indicators is of great significance for reducing the phenomenon of being prominently below the critical value and ensuring safe production. The fixed-size desorption index K_1_ is one of the important indicators for coal face and gas outburst prediction. Based on the diffusion theory and the physical meaning of fixed-size coal samples, the mathematical expression of K_1_ is established by the self-developed high/low temperature pressure swing adsorption-desorption experimental system. According to the equation, the effects of gas pressure, loss time, coal particle size and diffusion coefficient on K_1_ are studied. The results show that the K_1_ index is logarithmically related to the gas pressure. Under the same conditions, the longer the loss time is, the smaller the measured K_1_ is, and the smaller the particle sizes of the drill cuttings are, the more notable the performance is; the diffusion coefficient represents the ability of gas to bypass micropores and the coal matrix. The greater the ability to bypass the matrix is, the larger the diffusion coefficient under the same conditions is, and the larger K_1_ is; the coal particle size has a greater influence on K_1_, and the smaller the size is, the more likely it is that the phenomenon of being prominently below the critical value occurs. Therefore, the particle size composition of coal during on-site measurements is crucial for obtaining the true K_1_ and the exact critical values.

## Introduction

Coal and gas outbursts are serious natural disasters in coal mining that seriously threaten the safety of coal miners and result in property losses of coal mine enterprises^1^. Hazard prediction of the working face is an important part of preventing coal and gas outbursts. On-site and theoretical analyses show that^2^ a prominent coal seam not only has a higher gas content but also a higher gas desorption rate^3^.

The gas desorption index K_1_ is a widely used coal and gas outburst prediction index in China and is a fixed-size coal sample gas desorption index value^2^. The index is closely related to the desorption-migration law of gas in coal seams. Therefore, studying the law of gas desorption and diffusion of fixed-size coal samples is of great significance for safe coal mine production and coalbed methane mining.

Domestic and foreign scholars have performed much research on the laws of gas desorption and diffusion of fixed-size coal samples. Qin^4^ believes that gas in the adsorption state hardly participates in flow, while gas in the free state is the main body of gas flow, and the gas flow velocity is proportional to the pressure gradient, that is, the gas flow velocity conforms with Darcy’s law. Alexeev *et al*.^5^ divided the gas migration velocity of coal seams into two parts, diffusion velocity and seepage velocity, established a mathematical model of coal seam gas migration, and studied the gas dissipation law of coal seams. Since coal pores are mainly micropores and small pores with diameters of less than 10^−7^ m, most scholars have proposed that the gas migration velocity in coal is proportional to the gas content gradient based on a large number of gas desorption experiments. It is considered that the diffusion law can be improved by studying the gas desorption-migration law of coal samples. Yang *et al*.^6–9^ and other experts and scholars, based on Fick’s law of diffusion, established fixed-size coal gas diffusion models; Jia *et al*.^10–15^ conducted experiments to study the temperature and the influences of factors such as particle size, moisture and metamorphism degree on the law of gas diffusion. Yi *et al*.^16^ established a dual-medium model of gas desorption and diffusion in raw coal based on Fick’s law in porous media.

Most predecessors studied the gas diffusion of coal dust based on the classical diffusion model with a constant diffusion coefficient. The structures of coal and rock are complex, and the relationship between the diffusion coefficient and time is also very complicated. It is necessary to discuss the time response law of the diffusion coefficient. Therefore, based on the results of gas diffusion experiments with fixed-size coal samples, a gas diffusion model with the diffusion coefficient changing with time is established, and the theoretical solution of the time-varying diffusion coefficient of fixed-size coal samples is obtained by using the separation variable method. The research results enrich and improve the theory of gas desorption and diffusion of fixed-size coal samples and provide a reference for studying the gas emission law and coal and gas outburst mechanisms.

In addition, desorption index K_1_ can reflect the gas pressure of coal and the rate of gas desorption, so the index has become one of the predictors of the working surface widely used in mining works^2^. Many scholars have studied desorption index K_1_. Wu^3^ obtained the relationship between the gas pressure and the K_1_ index through experiments. Shao^17^ studied the effects of exposure time, number of measurements, and measurement time on K_1_. Zhao and Liu^18^ studied the effects of sampling, drilling holes, and instrument operation on K_1_. Due to the limitation of actual conditions, only the influence of the gas pressure on K_1_ was studied under the test conditions. However, the influence factors of K_1_ under field conditions can only be qualitatively analyzed; K_1_ is difficult to quantify, and is highly affected by the outside world. In short, there is a lack of comprehensive and in-depth theoretical analysis of K_1_ and its influencing factors.

According to the diffusion theory and the physical meaning of desorption index K_1_, the mathematical expression of K_1_ is derived. The influences of gas pressure, loss time, coal particle size and diffusion coefficient on K_1_ are studied by this equation to accurately determine K_1_ and improve the accuracy of hazard prediction.

## Experimental

### Experimental principle

The coal gas desorption index K_1_ of coal cuttings characterizes the gas desorption characteristics of coal samples. Previous experiments^19–22^ have shown that K_1_ is closely related to coal seam gas pressure, gas desorption rate, gas content, etc. The index has a certain functional relationship with the gas pressure and gas content and can reflect the magnitude of the prominent danger ahead of the working face. Therefore, this indicator can be used as an indicator of the outstanding risk.

In the determination of K_1_, the gas desorption law of coal samples can be considered to obey Eq. (1):

1 $$Q={{\rm{K}}}_{1}\sqrt{t}$$

where *Q* is the cumulative desorption of gas per unit mass of the coal sample from exposure time to time *t*, ml/g; *t* is the exposure time of the coal sample, min; and K_1_ is the gas desorption index of the drill cuttings, ml/(g·min^0.5^).

The movement of gas in coal is generally divided into three stages^6^: stage I, seepage in large pores controlled by the pressure gradient; stage II, diffusion process controlled by the concentration gradient in micropores; and stage III, gas desorption on the inner surfaces of the coal matrix.

Gas is physically adsorbed on the surface of coal, and the process of desorption is considered to be instantaneous. Seepage has little effect on granular coal. The latter is mainly manifested in the coal seam porosity and gas permeability measured by the usual method and is not directly related to the gas desorption rate of coal. Therefore, the process of gas escaping from drill cuttings mainly depends on the diffusion process, and the diffusion theory can be used to describe the law of gas emission after the coal cuttings or the drill cuttings fall off^7,8^.

The gas escaping from coal particles is due to the diffusion of gas in porous media, and its principle of emission is consistent with the diffusion law of Fick:

2 $$J=-\,D\frac{\partial {\rm{\rho }}}{\partial {\rm{x}}}$$

where *J* is the diffusion velocity, g/(s·m^2^); $\frac{\partial {\rm{\rho }}}{\partial {\rm{x}}}$ is the mass concentration gradient; *D* is the diffusion coefficient, m^2^/s; and *ρ* is the mass concentration of the diffusion fluid, g/m^3^.

Assumptions: 1) The coal particles are spherical; 2) the coal particles are homogeneous and isotropic; 3) the gas flow follows the principles of mass conservation and continuity. Then, the second law of diffusion in the spherical coordinate system can be obtained:

3 $$\frac{\partial {\rm{\rho }}}{\partial {\rm{t}}}=D(\frac{{\partial }^{2}{\rm{\rho }}}{\partial {{\rm{r}}}^{2}}+\frac{2}{r}\frac{\partial {\rm{\rho }}}{\partial {\rm{r}}})$$

where *r* is the polar radius of the coal particles, m.

Solving Eq. (3) results in^9^:

4 $$\frac{{Q}_{t}}{{Q}_{\infty }}=1-\frac{6}{{\pi }^{2}}{\sum }_{n=1}^{\infty }\frac{1}{{n}^{2}}{e}^{-{n}^{2}\frac{{\pi }^{2}D}{{d}^{2}}t\times 60}$$

In the equation, *Q*_t_ is the gas desorption amount accumulated when the gas desorption time is *t*, m^3^/t; *Q*_∞_ is the limit gas desorption amount when the gas desorption time approaches infinity, m^3^/t; *d* is the coal particle radius, m; and *n* is a natural number (1, 2, 3… *n*).

According to the Langmuir equation, the coal seam gas content can be expressed as:

5 $${\rm{W}}=\frac{abP}{1+bP}\frac{1}{1+0.31{M}_{ad}}\frac{(100-{A}_{ad}-{M}_{ad})}{100}+\frac{10\lambda P}{{\gamma }_{a}}$$

where *a* is one of the adsorption constants, which represents the maximum amount of gas adsorption, that is, the maximum amount of adsorbed gas when the gas pressure tends to infinity at a certain temperature, also known as the Langmuir volume, cm^3^/g; b is another adsorption constant, which represents the reciprocal of the Langmuir pressure, MPa^−1^; *P* is the gas pressure of the coal seam, MPa; *M*_ad_ is the air-dried basis moisture content, %; *A*_ad_ is the air dry base ash content, %; *λ* is the porosity of coal, %; and *γ*_a_ is the apparent relative density, g/cm^3^.

Therefore, under standard atmospheric conditions, the maximum gas desorption amount *Q*_∞_ can be expressed as:

6 $${Q}_{\infty }=(\frac{abP}{1+bP}-\frac{0.1ab}{1+0.1b})\frac{1}{1+0.31{M}_{ad}}\times \frac{(100-{A}_{ad}-{M}_{ad})}{100}+(10P-1)\frac{\lambda }{{\gamma }_{a}}$$

The mathematical expression of gas desorption index K_1_ of the drill cuttings can be derived from Eqs (1), (4), and (6). That is:

7 $$\begin{array}{rcl}{K}_{1} & = & (1-\frac{6}{{\pi }^{2}}\mathop{\sum }\limits_{n=1}^{\infty }\frac{1}{{n}^{2}}{e}^{-{n}^{2}\frac{{\pi }^{2}D}{{d}^{2}}t\times 60})\\  &  & \times [(\frac{abP}{1+bP}-\frac{0.1ab}{1+0.1b})\frac{1}{1+0.31{M}_{ad}}\frac{(100-{A}_{ad}-{M}_{ad})}{100}+(10P-1)\frac{\lambda }{{\gamma }_{a}}]/\sqrt{t}\end{array}$$

where the parameter indicators in Eq. (7) have the same meaning as above.

### Experimental equipment

**Figure 1:**
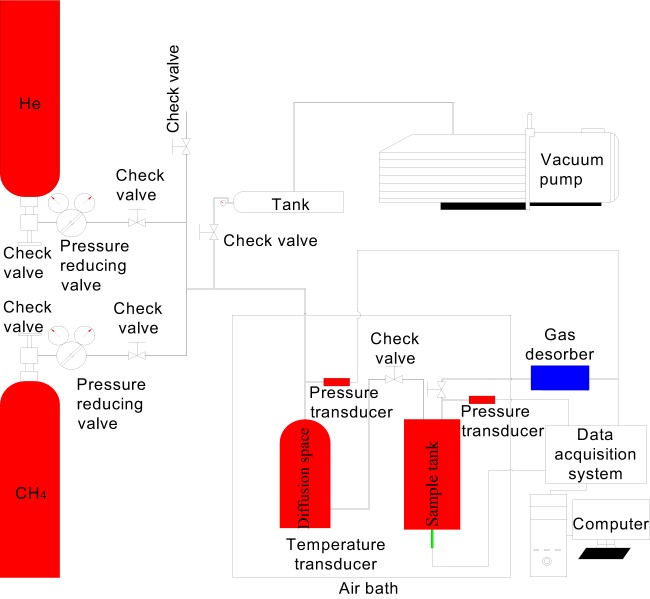
Schematic diagram of the experimental platform for the high-low temperature coal gas desorption and adsorption experiments^23^.

The self-developed high/low temperature pressure swing adsorption-desorption experimental system^23^ is shown in Fig. 1. The experimental equipment mainly consists of an adsorption system, a gas desorption system, a refrigeration system, an electrical control system, and a computer data acquisition and processing system.

The components of the experimental platform are as follows:

1. The temperature variation range of the high and low temperature variable frequency control system is −50 °C to 50 °C, the temperature deviation is ≤ ±1 °C, and the temperature fluctuation is ±0.5 °C;
2. The adsorption balance unit is composed of a precision pressure sensor, gas with a pressure of 15 MPa and purity of 99.9% (vol/vol), reference tank, sample tank, and various control valves. The multifunctional coal sample tank can measure the gas desorption of coal in real time and the temperature change during adsorption;
3. The vacuum degassing unit is composed of a resistance vacuum gauge (1 − 1 × 10^5^ Pa), a vacuum pump (extreme vacuum degree 6.8 × 10^−2^ Pa), a vacuum tube and a three-way valve;
4. The desorption measurement control unit is composed of a pressure regulating valve and a gas desorption measuring instrument. The pressure regulating range of the pressure regulating valve is 0-16 MPa, and the pressure regulating scale is 0.005 MPa/dial gauge unit, and the gas desorption measuring instrument is composed of a descaling measuring cylinder with a scale. The inner diameter of the measuring tube is 50 mm, the height is 500 mm, the volume is 1000 ml, and the minimum precision is 2 ml;
5. The real-time data acquisition system transmits the test data of the experiment to the data acquisition system through the temperature and pressure sensors, and the data acquisition system is connected with the computer to realize the real-time collection of the test data.

### Sample preparation

The experimental coal sample is coking coal taken from the No. 4 coal seam of the Nantong coal mine in Chongqing. Each recoverable coal seam is judged to be in danger of coal and gas outbursts according to the critical thresholds of four indicators for the identification of outburst-prone coal seams (as shown in Table 1)^24^. After fixed-size sampling of the coal seam, the coal sample is processed and industrially analyzed according to the Proximate Analysis Method of Coal (GB/T212-2008)^25^. The industrial analysis results are shown in Table 2. The coal sample is crushed according to the standards, and the coal sample with a particle size of 1 to 3 mm is sieved. Using a drying apparatus, the coal sample is dried at 105 °C for 4 h and placed in a ground jar for use. Because the experiment aims to study the coal gas desorption and adsorption law of coal briquettes at different forming pressures, the preparation of briquette samples should be carried out according to the experimental design of the water and molding pressures during the preparation of briquette samples.

**Table 1:** Thresholds of four indicators for the identification of outburst-prone coal seams.

Term	*D*_cf_	Δ*P* (mm Hg)	*f*	*P* (MPa)
Thresholds	III, IV, V	≥10	≤0.5	≥0.74

Notes: *D*_cf_ is the degree of coal fracturing, dimensionless; Δ*P* is the initial velocity of diffusion of coal gas, mm Hg; *f* is the coal hardiness coefficient, dimensionless; and *P* is the measured gas pressure in the coal seam, MPa.

**Table 2:** Results of the proximate analysis of the coal samples.

*M*_ad_ (%)	*A*_ad_ (%)	*V*_daf_ (%)	*a* (cm^3^/g)	*b* (MPa^−1^)	*γ*_t_ (g/cm^3^)	*γ*_a_ (g/cm^3^)
1.60	18.66	12.12	34.639	0.686	1.51	1.48

*M*_ad_ is the air-dried basis moisture content, %; *A*_ad_ is the air dry base ash content, %; *V*_daf_ is the dry ashless basis volatile matter, %; *a* and *b* are Langmuir adsorption constants, cm^3^/g, MPa^−1^; *γ*_t_ is the true relative density, g/cm;^3^ and *γ*_a_ is the apparent relative density, g/cm^3^.

**Figure 2:**
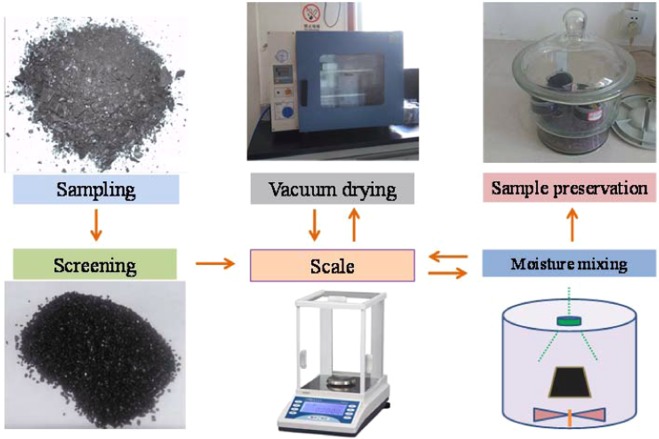
Humidification process for the coal samples.

The prepared coal samples with different moisture contents need to be manually humidified after the coal samples are dried. A schematic diagram of the coal humidification treatment device is shown in Fig. 2. The water in the distillation bottle is heated by a heater to distill the water. The water is mixed with the coal sample to obtain the water-saturated coal sample, and then the coal sample is dried to different degrees according to the literature^26^ or the vacuum degassing method is used to obtain a coal sample with a set moisture content; then, a small coal sample amount is selected. Drying and weighing are used to measure the moisture content, and the moisture of the obtained coal sample is examined.

### Experimental methods

Coal sample gas desorption process simulation was conducted by employing the experimental device shown in Fig. 1. The gas desorption environment of the sample was always maintained at a temperature of 30 ± 1 °C and a gas outlet pressure of 0.1 MPa during the measurement process, and gas desorption of the coal sample could be considered to be an isothermal and isostatic desorption process. The experiment is conducted following the steps below:

1. The experimental coal sample is loaded into the coal sample tank.
2. The coal sample tank and the reference tank are fully degassed using a vacuum pump.
3. When the vacuum in the experimental system reaches 25 kPa, gas is introduced into the reference tank. When the pressure is constant, the coal sample tank and the reference tank are connected to conduct gas adsorption.
4. As gas adsorption balance in the coal sample is reached, the gas inlet valve is first closed, and the gas outlet valve is opened second to conduct the gas desorption experiment.
5. After the experiment is completed, the measured gas desorption amount is converted into volume in the standard state.

## Results and Analysis

**Figure 3:**
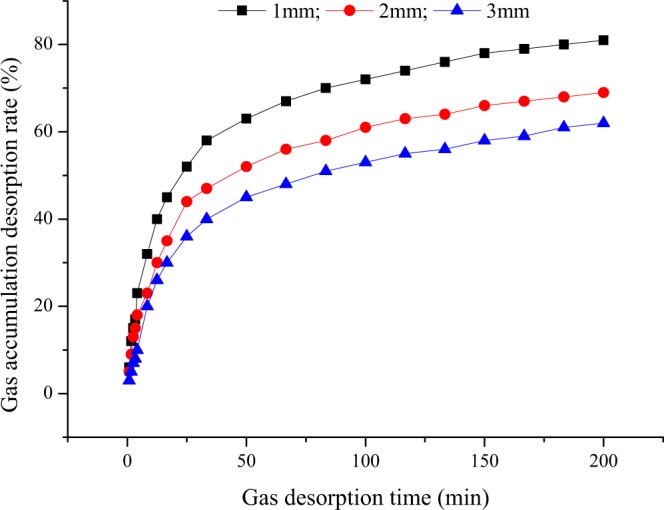
Gas cumulative desorption rate curve.

According to the measured desorption amount of the experimental coal sample with time, the cumulative gas desorption rate (*Q*/*Q*_∞_) of the experimental coal sample is calculated. The change trend with time is obtained, as shown in Fig. 3.

Figure 3 shows that:

1. At the initial stage of coal gas desorption, the coal sample desorbs rapidly, and the cumulative desorption rate increases sharply. As the gas desorption time is extended, the gas desorption rate of the experimental coal sample gradually decreases.
2. The cumulative desorption rate of gas in the experimental coal samples increases with extension of the desorption time, which is manifested in the cumulative desorption rate curve of coal sample gas that increases with time.
3. The cumulative desorption rate of coal sample gas with time is a monotonically increasing function with an upper limit.

Function fitting of the cumulative gas desorption rate data shows that the optimal function is a logarithmic function. The optimal fitted function equation of the cumulative gas desorption rate of the coal samples with different particle sizes is shown in Table 3.

**Table 3:** Optimal fitting equation of the gas cumulative desorption rate.

Particle size	Optimal fit equation	R^2^
1 mm	*r*_ad_ = 14.836Ln(t) + 3.4198	0.9941
2 mm	*r*_ad_ = 12.726Ln(t) + 1.4555	0.9899
3 mm	*r*_ad_ = 12.024Ln(t) − 2.7208	0.9864

Notes: *r*_ad_ is the gas cumulative desorption rate, %, and R^2^ is the adjusted R-square value, dimensionless.

Table 3 shows that the gas cumulative desorption rate of the fixed-size coal sample changes with time according to a logarithmic function, and the correlation coefficient of the fitted equation is greater than 0.9864. Moreover, the larger the coal sample particle size is, the worse the correlation. However, because the correlation is higher, the fitted function can represent the trend of the measured data.

## Discussion

### Effect of the gas pressure on desorption index K_1_

**Figure 4:**
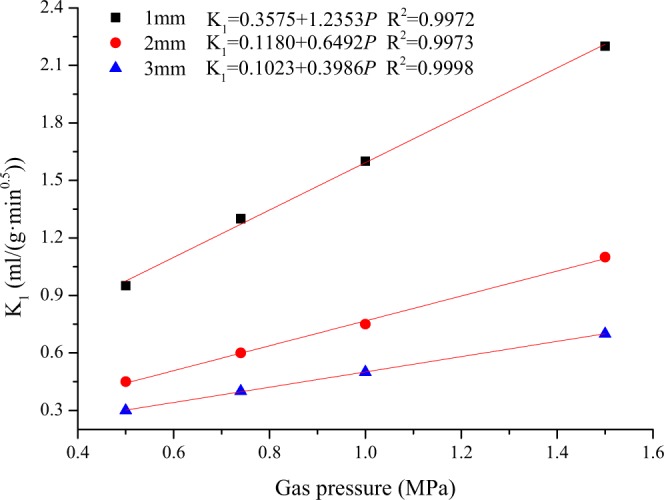
Relationship between gas desorption index *K*_1_ and gas pressure.

Take the diffusion coefficient D = 1 × 10^−10^ m^2^/s for n = 100 and substitute the index of Table 1 and t = 1 min into Eq. (7) to obtain the change in K_1_ with the gas pressure under different particle size conditions (as shown in Fig. 4). The gas desorption index K_1_ and gas pressure are linearly related^27^, which can be expressed as:

8 $${{\rm{K}}}_{1}={\rm{a}}+{\rm{b}}P$$

**Figure 5:**
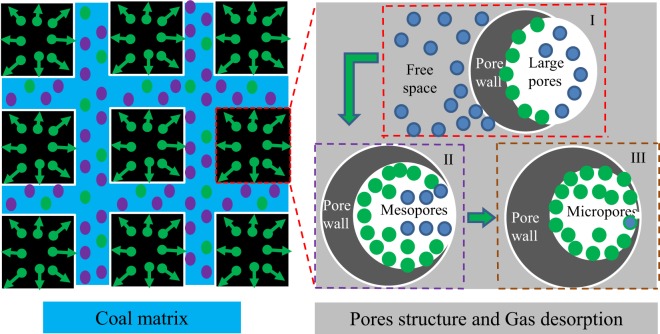
Schematic diagram of the gas diffusion process in the coal matrix.

This equation shows that K_1_ can reflect the magnitude of the gas pressure well. However, as the coal particle size decreases, gas desorption index K_1_ increases significantly under the same gas pressure conditions. K_1_ represents the gas desorption of a fixed-size coal sample 1 min after pressure relief. Assuming that the coal particles are cubic (see Fig. 5), the higher the gas pressure inside the coal cuttings is, the higher the gradient of the coal cuttings and external gas concentration are. Therefore, the measured K_1_ under the same conditions is larger.

During the desorption process, the free space and large pores in the coal are first desorbed and diffused^28,29^. Since this part of the gas is in the free state, the intermolecular binding force is small, and the desorption process has a high gas desorption rate. However, with the diffusion of free gas, the coal sample gas desorption rate decays rapidly, corresponding to stage I in Fig. 5. This stage is the desorption process of free gas and part of the adsorbed gas in the pores of the coal body, which is accompanied by shrinkage of the pore structure.

Desorption and diffusion of the adsorbed gas continues, the volume of the coal body shrinks, the pore size decreases, and the desorption process of gas is gradually affected by the capillary hole resistance, corresponding to stage II of Fig. 5, which is the desorption process of coal pore-adsorbed gas, accompanied by shrinkage of the pore structure.

As the desorption process progresses, in stage III, the gas desorption process involves more of the micropore structure in which methane molecules are hydrogen-bonded to the oxygen-containing functional groups on the coal surface, and the desorption of methane molecules is controlled by adsorption/desorption. Corresponding to stage III in Fig. 5, where the pores of the coal body are gradually closed and the amount of gas desorption at atmospheric pressure is gradually reduced.

### Effect of the gas desorption time on desorption index K_1_

**Figure 6:**
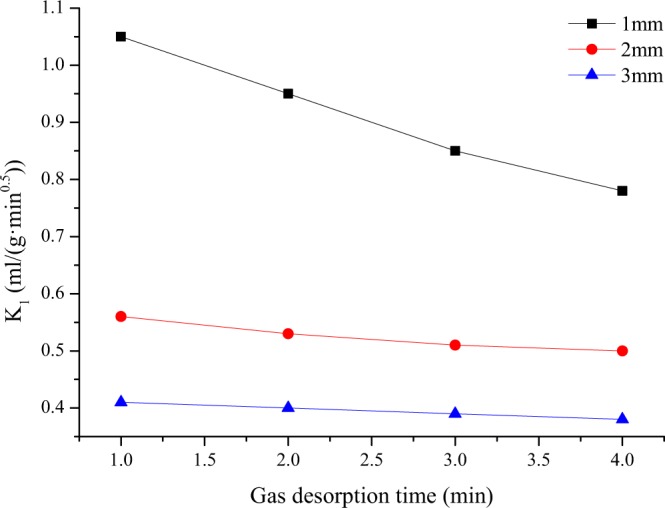
Relationship between the gas desorption index and gas desorption time.

At an equilibrium gas pressure of 0.74 MPa, the relationship between gas desorption index (K_1_) and gas desorption time of the coal sample is shown in Fig. 6. It can be seen from Fig. 6 that under the same gas pressure conditions, when the coal particle size is 3 mm and 2 mm, K_1_ decreases with the prolonged free desorption time of the coal sample, but the degree of decrease is low. When the coal sample size is 1 mm, K_1_ decreases with the desorption time, and the particle size decreases by more than 1 mm. However, when the coal sample size is 1 mm, K_1_ of the fixed-size coal sample decreases significantly. The smaller the coal particle size is, the more notable the effect of the desorption time on K_1_ is, and the smaller the measured K_1_ is compared to the actual value. Therefore, the free desorption time of the coal sample should be minimized during the on-site determination of K_1_. Especially for the construction of coal, the higher the probability of failure is, the smaller the particle size of the coal being crushed by the crucible, and the longer the corresponding free desorption time. Then, the measured K_1_ is smaller than the actual value, so that the phenomenon of being prominently below the critical value is likely to occur^30–32^.

### Effect of diffusion coefficient on desorption index of K_1_

**Figure 7:**
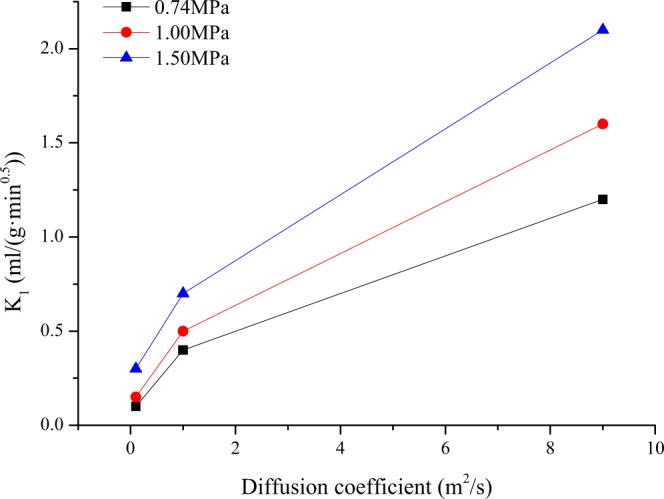
Relationship between gas desorption index K_1_ and diffusion coefficient.

**Figure 8:**
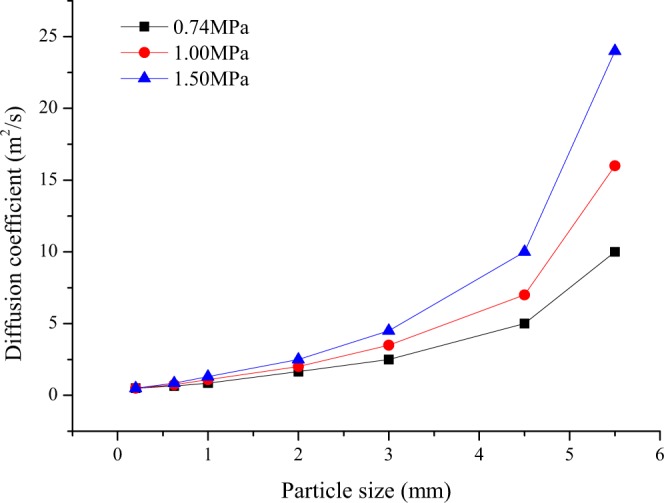
Relationship between the diffusion coefficient and particle size at different gas pressures.

The variation in gas desorption index K_1_ with the diffusion coefficient is shown in Fig. 7. With the increase in the diffusion coefficient, K_1_ shows an increasing trend; the larger the diffusion coefficient is, the more notable the change in K_1_ is under different pressure conditions. The diffusion coefficient characterizes the ability of gas to bypass the coal matrix and micropores to enter the outside of the coal cuttings^23,33–35^. As shown in Fig. 8, the larger the diffusion coefficient and the higher the gas pressure are, the larger the corresponding K_1_. However, when the gas pressure is 0.74 MPa, K_1_ deviates from its critical value of 0.5 ml/(g·min^0.5^) due to the difference in the diffusion coefficient.

### Effect of the particle size on desorption index K_1_

**Figure 9:**
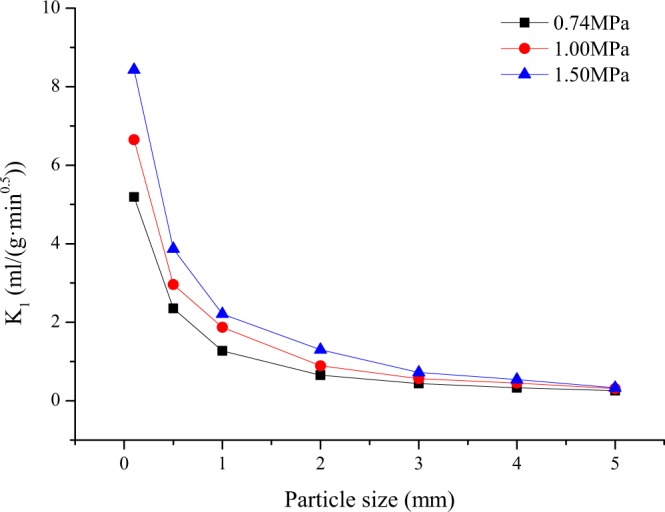
Relationship between K_1_ and particle size at different gas pressures.

Under different gas pressure conditions, the change in gas desorption index K_1_ with the coal particle size is shown in Fig. 9. Under the same gas pressure conditions, with the increase in coal particle size, K_1_ shows a trend of rapid decline. The larger the coal particle size is, the smaller K_1_ is under different gas pressure conditions^36–38^. As seen in conjunction with Fig. 9, the larger the particle size of coal is, the longer the path of gas bypassing the micropores and the coal matrix is, and in addition, the less likely gas is to emerge from the drill cuttings; hence, the measured K_1_ is smaller.

D = 1 × 10^−10^ m^2^/s and K_1_ corresponding to several coal particle sizes at 0.74 MPa is shown in Table 4. Table 4 shows that the smaller the coal particles are, the more intense K_1_ changes with the particle size. When the coal particle size is smaller than 1 mm, a smaller particle size change will cause a large change in K_1_; for example, when the particle size is 0.5 mm, K_1_ is 1.85 times K_1_ when the particle size is 0.1 mm, and K_1_ for a particle size of 0.1 mm is 2.21 times K_1_ for a particle size of 0.5 mm; the larger the particle size is, the gentler the effect of the particle size change on K_1_ tends to be. When the particle size is larger than 3 mm, K_1_ for a particle size of 4 mm is 1.33 times the K_1_ corresponding to a particle size of 3 mm, and K_1_ for a particle size of 4 mm is 1.26 times K_1_ for a particle size of 5 mm. As a predictive indicator of the working surface, K_1_ must be related to coal and gas dynamics phenomena and have a certain degree of change. Therefore, it is more appropriate to select a range of 1–3 mm as the K_1_ particle size. However, even in the range of 1–3 mm, the difference in particle size has a greater impact on the results.

**Table 4:** Gas desorption index K_1_ for different particle sizes.

Particle size (mm)	0.1	0.5	1	3	4	5
K_1_ (ml/(g·min^0.5^))	5.19	2.35	1.27	0.44	0.33	0.26

The experimental adsorption equilibrium gas pressure is 0.74 MPa.

**Figure 10:**
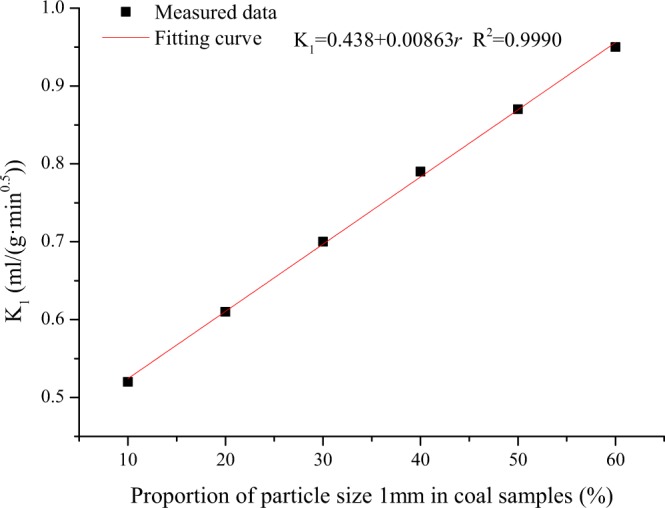
Relationship between K_1_ and the 1 mm particle size proportion in coal.

For example, when the coal particle size is 1 mm at 0.74 MPa, K_1_ is 2.88 times K_1_ when the coal particle size is 3 mm. At the given gas pressure, K_1_ has a strong relationship with the coal particle size composition. Figure 10 reflects the influence of the proportion of the total coal sample with a 1 mm particle size on K_1_. In the on-site measurement process, special attention should be paid to the screening process to prevent excessive particle size changes.

## Conclusions

In this paper, through on-site sampling, the self-developed high/low temperature pressure swing adsorption-desorption experimental system is used to measure the gas desorption index of fixed-size coal samples under different pressure conditions, and the test results have been theoretically analyzed and discussed. Finally, the following conclusions have been drawn:

1. The gas desorption index K_1_ can reflect the magnitude of the gas pressure well, and K_1_ and gas pressure have a logarithmic functional relationship.
2. With increasing free desorption time, K_1_ has a decreasing trend. The smaller the coal particle size is, the more notable the impact of the free desorption time on gas desorption index K_1_. In particular, for structural coal, the longer the free desorption time is, the lower the measured K_1_ is relative to the actual value. The smaller the size is, the more likely it is that the phenomenon of being prominently below the critical value occurs.
3. The gas desorption index K_1_ has a strong relationship with the coal particle size composition. During the on-site measurement process, special attention should be paid to the screening process to prevent excessive particle size changes.
4. The diffusion coefficient is a very important parameter when determining the threshold of gas desorption index K_1_ and should be fully co*ns*idered^39,40^.
